# Supplementary material for: Proportion and seasonality of blood parasites in animals in Mosul using the Veterinary Teaching Hospital Lab data
Source: PLoS One. 2022 Feb 22;17(2):e0264121. doi: 10.1371/journal.pone.0264121 (PMC8863285; doi:10.1371/journal.pone.0264121)
Supplement: Attachment — Submitted filename: Response to Reviewers.docx [file pone.0264121.s001.docx]

**Manuscript ID: PONE-D-21-39858**

**Title: “Proportion and Seasonality of Blood Parasites in Animals in Mosul using the Veterinary Teaching Hospital Lab Data”**

-------------------------------------------------------------------

**Bold text indicates questions, comments, or concerns expressed by the reviewer.**

AU: the author.

Regular text indicates the authors’ response.

~~Strikethrough text indicates deleted text.~~

Underlined text indicates new text.

*Text in Italics indicates text as presented in the revised manuscript.*

--------------------------------------------------------------------

**REVIEWER # 1**

**This is an interesting epidemiological survey that provides new insights into the seasonality of infections caused by blood-borne pathogens in the animals of Mosul. I only have some minor suggestions for improvement, which are below, but other than that, I think it largely reads well!**

AU: The authors thank the reviewer for the review and critique of this manuscript. As a result, the authors have produced a better manuscript.

**1. In general, I would suggest changing "blood parasites" to "blood pathogens" or something that covers bacteria as Anaplasma, Ehrilichia, etc are not specifically parasites, even though some consider them as such.
I would also italicize any "et al" when referencing authors.**

AU: Yes, the authors agree with the reviewer. However, Tylor *et al*., (2016) mentioned that although Rickettsia are now considered to be in the kingdom Bacteria, they are included in parasitological books for historical reasons. In that book:

“Species of Anaplasma are very small (0.3–1.0 μm diameter) parasites of the erythrocytes of ruminants and are transmitted biologically by ticks and mechanically by sucking flies”.

Taylor, Coop, R. L. & Wall, R. (2016). Veterinary parasitology. 4^th^ ed. Wiley Blackwell.

In addition, the authors follow PLOS ONE guidelines concerning italicizing the “et al”.

**2. In your title, it may beneficial to specify what animals you examined, maybe "...livestock and dogs in Mosul..."?**

AU: We haven’t specified animals because all animals were considered for inclusion. This was mentioned in the material and method section, Lns 84-86:

*Animals examined at the University of Mosul Veterinary Teaching Hospital clinical pathology laboratory for evidence of blood parasites in blood smears were considered for inclusion in this study.*

**3. In line 45 of your introduction, it should say "blood parasitic (or pathogenic if you change it) infections constituted..." and in line 68, it should say "such as those by Al-Obaidi...".**

AU: Done!

**4. In your methods, I would recommend mentioning what animals you examined or refer to Table1.**

AU: Done! Please, see revised manuscript, Lns: 86-87:

*These animals included cattle, sheep, goats, buffaloes, horses, and dogs.*

**In line 90, it should be "tick infestations" and in your Laboratory Examination paragraph, it should read "*Trypanosoma* spp. were intercellular..." and the same for the other pathogens. In line 109-110, it should say "...and either single or mixed...".**

AU: Done!

**I am also just curious, was there a reason the abundance (or number) of parasites/bacteria in each blood smear was not examined?**

AU: A quantitative examination was not performed for all blood smears. Therefore, parasitaemia was ignored in this study. It is an option for further investigations.

**5. In your results, I would add the total number of samples examined between "...341 blood smears" and "(61.77%)...".**

AU: Done! Please, see revised manuscript, Lns: 133-134:

*~~A total of 341 blood smears~~ Among 552 blood smears were examined, 341 (61.77%) were tested positive for blood parasites from different animals.*

**Line 143-144 should say "Additionally, the differences in the proportion...".**

**I would also only have "...at the Veterinary Teaching Hospital | University of Mosul between Oct 30, 2017 to Dec 31, 2019" on the first caption rather than each one.**

**Also can you explain what "Referent" means under 95% CI in Table 2?**

AU: Done!

**6. In your discussion, line 198 should read "...city to examine..." and line 201 should say "...blood parasites".**

AU: Done! Please, see revised manuscript, Lns 199-200:

*~~This~~ The current study is considered the first study in Mosul city that examined the seasonality of blood parasites in animals*

**In line 202, I would maybe change "burden" to "infection rate" as that sounds more like abundance.**

**In line 229-230, I would change it to read "[6, 23], Babesia ovis, B. motasi, B. foliate, and B. taylori in goats [14, 25]...".**

**Line 239-241 would be better as "...Anaplasma marginale has been identified in buffalo [27], camel [28] while Anaplasma ovis was detected in goats [5, 14] in addition to that reported in cattle...".**

**There should be an author referenced in line 242 and 249 after "for [2]" and "of [2]' and some references in line 247 after "...camels".**

**Line 252 should be "adult animals..."**

AU: Done!

**In line 253, it should be "On the other hand, nineteen..."**

**Line 254 should read "...animals [reference]; however,..."**

AU: Done. Please, see revised manuscript, Lns 257-259:

*~~Under our hands nineteen~~ The current study cited eighteen local studies examined blood parasites in different animals [2-7, 14,15, 20-29], however; none of them have reported the difference*

**Line 258, it should be "...adults as well as young...".**

AU: We prefer to keep it as it in the manuscript as we have compared the percentages between adult and young animals.

**7. In your conclusion, I would again change "...substantial burden" to something like "a relatively high infection rate" in line 274.**

AU: Done!

**Line 276-277 should be "...endemic understanding of the infection..."**

AU: Please, see revised manuscript, Ln 282:

*contribute to the endemic ~~picture~~ situation of the infection in the city.*

**8. Your tables and figures mostly look good but in Figure 3, Theileria is misspelled and I wonder if it also needs standard error/deviation bars?**

AU: Done!

Standard error/deviation bars were not added to avoid confusion.

**I hope these suggestions help make minor improvements but it is a very fascinating paper otherwise!**

AU: Thank you very much, we appreciate your inputs!

**REVIEWER # 2**

**Minor Issues:
• Formatting issues (figures do not line up with the formatting e.g. Page 8, Fig 1. Page 9, Fig 2 and Page 11, Fig 3.)**

AU: We will work with the editor on this issue. Thanks!

**• There is no mention of n (total sample size) analysed within the study animals/data collection section.**

AU: We agree. The number of examined animals was added in the results section according to the comment of the reviewer #1. Please, see revised manuscript, Lns: 133-134:

*~~A total of 341 blood smears~~ Among 552 blood smears were examined, 341 (61.77%) were tested positive for blood parasites from different animals.*

**• Page 12, line 209: ‘it seems that this infection has emerged to the city around 2011’, this statement has no reference or further information to support the claim being made.**

AU: Done! Please, see revised manuscript, Lns 210-214:

*It seems that this infection has emerged to the city around 2011 as a result of uncontrolled importation [15, 20, 21], and ~~spread~~ then widely spread between local animals to become the most frequent blood parasite infection diagnosed in blood smears in the current investigation.*

**• Page 11, line 198, this does not read correctly and has grammatical issues.**

AU: Done! Please, see revised manuscript, Lns 199-200:

*~~This~~ The current study is considered the first study in Mosul city that examined the seasonality of blood parasites in animals*

**• Page 12, line 201 ‘relatively considered high’, requires rewording.**

AU: Done! Please, see revised manuscript, Ln 203:

*is considered relatively ~~considered~~ high*

**• Page 14, line 235 ‘in Mosul in animal has’, grammatical issue.**

AU: Done! Please, see revised manuscript, Ln 237-238:

*It is difficult to consider that the prevalence of Babesia spp. ~~in Mosul~~ in animals in Mosul city has decreased*

**• Page 15, line 260 ‘examined’ requires grammatical correction.**

AU: Done! Please, see revised manuscript, Ln 266:

*study is considered the first local study that examined the seasonality*

**• Page 11, line 179 ‘increase in the in’ grammatical issue.**

AU: Done!

**• Page 10, line 170 ‘the proportion of blood parasites infection’**

**Major Issues:

• Criterion of examining certain blood samples only from symptomatic (icteric mucous membranes, enlargement of superficial lymph nodes, haemoglobinuria, ticks, and prolonged illness) animals, has potentially limited the true reflection of parasitic infection prevalence within Mosul; therefore also limiting the true reflection of seasonal parasitic infections as only clinically unwell animals are examined microscopically due to circumstances of being brought to the Veterinary Hospital and blood taken under these animals meeting these conditions. Low burden parasitic infections can be asymptomatic, and equally, parasites are still found in clinically healthy animals, including cattle (example:** [**https://parasitesandvectors.biomedcentral.com/articles/10.1186/s13071-016-1498-1**](https://parasitesandvectors.biomedcentral.com/articles/10.1186/s13071-016-1498-1)**).**

AU: The authors totally agree with the reviewer, and for this reason we did not use the term “prevalence” in our report. The investigation targeted only sick animals.

Finally, we are trying to improve our data collected at the veterinary teaching hospital; therefore, studies are conducted to explore gaps in this data.

**• Total sample size is 552 animals. A larger sample size may have benefited this study and increased reliability of results and therefore the conclusions that can be drawn from a larger data set, by including animals that do not visually manifest a certain set of criteria. This can eliminate bias (only including animals manifesting certain symptoms) and more asymptomatic cases and low parasitic burdened animals could be found and may offer more data.**

AU: This is the available number in the records of the hospital. In our conclusion, we have suggested conducting more epidemiological studies.

**• The data used to ‘assign’ seasonality (Winter: 1st December to 28th February. Spring: 1st March to 31st May. Summer: 1st June to 30th September and Autumn: 1st October to 30th November) was stated as the examination date, not the sample collection date. This has the potential to be inaccurate due to possible delayed examinations and other circumstances that can delay the process of identification; unless otherwise stated that the examination date and collection date are equivalents, and do not differ. Is the examination date the same as the collection date of the original sample?**

AU: The examination date was the same as the collection date.

**Additional comments:

A thoroughly intriguing study and I enjoyed reading it. This research fills an important gap within knowledge concerning parasitic burden in animals within Mosul, and it is a key element that can not be overlooked for animal and human health, with good reason. I would be happy to review this paper again.**

AU: The authors thank the reviewer for the review and critique of this manuscript. As a result, the authors have produced a better manuscript.

**END**
